# *Schistosoma mansoni* Mucin Gene (*Sm*PoMuc) Expression: Epigenetic Control to Shape Adaptation to a New Host

**DOI:** 10.1371/journal.ppat.1003571

**Published:** 2013-08-29

**Authors:** Cecile Perrin, Julie M. J. Lepesant, Emmanuel Roger, David Duval, Sara Fneich, Virginie Thuillier, Jean-Francois Alliene, Guillaume Mitta, Christoph Grunau, Celine Cosseau

**Affiliations:** 1 Université de Perpignan Via Domitia, Perpignan, France; 2 CNRS, UMR 5244, Ecologie et Evolution des Interactions (2EI), Perpignan, France; 3 Center for Infection and Immunity of Lille, Inserm U1019, CNRS UMR 8204, Institut Pasteur de Lille, University Lille Nord de France, Lille, France; George Washington University School of Medicine and Health Sciences, United States of America

## Abstract

The digenetic trematode *Schistosoma mansoni* is a human parasite that uses the mollusc *Biomphalaria glabrata* as intermediate host. Specific *S. mansoni* strains can infect efficiently only certain *B. glabrata* strains (compatible strain) while others are incompatible. Strain-specific differences in transcription of a conserved family of polymorphic mucins (*Sm*PoMucs) in *S. mansoni* are the principle determinants for this compatibility. In the present study, we investigated the bases of the control of *Sm*PoMuc expression that evolved to evade *B. glabrata* diversified antigen recognition molecules. We compared the DNA sequences and chromatin structure of *Sm*PoMuc promoters of two *S. mansoni* strains that are either compatible (C) or incompatible (IC) with a reference snail host. We reveal that although sequence differences are observed between active promoter regions of *Sm*PoMuc genes, the sequences of the promoters are not diverse and are conserved between IC and C strains, suggesting that genetics alone cannot explain the evolution of compatibility polymorphism. In contrast, promoters carry epigenetic marks that are significantly different between the C and IC strains. Moreover, we show that modifications of the structure of the chromatin of the parasite modify transcription of *Sm*PoMuc in the IC strain compared to the C strain and correlate with the presence of additional combinations of *Sm*PoMuc transcripts only observed in the IC phenotype. Our results indicate that transcription polymorphism of a gene family that is responsible for an important adaptive trait of the parasite is epigenetically encoded. These strain-specific epigenetic marks are heritable, but can change while the underlying genetic information remains stable. This suggests that epigenetic changes may be important for the early steps in the adaptation of pathogens to new hosts, and might be an initial step in adaptive evolution in general.

## Author Summary

*Schistosoma mansoni* is a parasitic worm and agent of a disease that causes a considerable economic burden in African and South American countries. The propagation of the parasite requires passage through a freshwater snail of *Biomphalaria* genus. In the field, actually very few snails are infected. This is due to the fact that specific strains of the parasite can infect only specific strains of the snail. Comparative studies have shown that this so-called compatibility is based on the expression of a family of genes that are called *Sm*PoMucs. We have shown previously that all parasites strains possess the repertoire of all *Sm*PoMuc genes but every strain and even every individual parasite expresses only a subset. These differences could be due to DNA sequence differences in the regions that control gene expression, but here we show that these regions are nearly identical. Instead, the chromatin structure shows strain-specific characteristics. This means that the parasite can adapt to different snail strains simply by changing its chromatin structure and not necessarily the DNA sequence. If this holds true for other parasites, then we have to rethink the way parasite evolution is currently imagined but this also provides a new potential entry point to control the spread of diseases.

## Introduction

The interaction of hosts and parasites is one of the best-studied examples of evolution in a changing environment [1]. Their reciprocal antagonistic co-evolution can be illustrated by an arms race in which host and parasite develop mechanisms to circumvent counter-measures developed by their opponents [2], [3]. Under certain conditions, parasite virulence and host defence can be in equilibrium leading to a phenomenon called compatibility. Compatibility occurs in a host-parasite system when the parasite species is capable of infection and transmission through the host species [4]. The phenomenon that some parasite strains are compatible with certain host strains but not with others (and *vice versa*) is called compatibility polymorphism. This phenomenon was described in the platyhelminth *Schistosoma mansoni* and its intermediate host, the mollusc *Biomphalaria glabrata* [5]. *S. mansoni* is a human parasite whose life cycle is characterised by the passage through two obligatory sequential hosts: the fresh-water snail *B. glabrata* (or dependent on the geographical location other *Biomphalaria* species) for asexual replication, and humans or rodents as hosts for sexual reproduction [6]. The molecular mechanisms underlying compatibility polymorphism between *S. mansoni* and *B. glabrata* were recently investigated by comparing the proteomes of two *S. mansoni* laboratory strains: one strain that is compatible (the C strain) and one that is incompatible (the IC strain) with the same reference *B. glabrata* strain from Brazil [7]. The study identified *S. mansoni* Polymorphic Mucins (*Sm*PoMucs) as key markers for compatibility (see [4] for a recent review). *Sm*PoMuc glycoproteins have a mucin-like structure with an N-terminal domain containing a variable number of tandem repeats (VNTR) [8]. *Sm*PoMuc proteins are highly polymorphic [8] and interact with the Fibrinogen RElated Proteins (FREPs) of the mollusc [9]. FREPS are diversified antigen recognition molecules playing a central role in the secondary immune response to digenetic trematodes [10], [11], [12]. The extraordinary level of *Sm*PoMuc polymorphism is generated by a complex cascade of mechanisms, a “controlled chaos”, acting at the transcriptional, translational and post-translational level [8]. *Sm*PoMucs are encoded by a multigene family with at least 10 members that are organised in 4 clusters on the genome. They recombine frequently and generate new alleles [8]. Each individual miracidium (the larva that infects the mollusc) expresses only a specific subset of *Sm*PoMuc genes. The mechanisms controlling this expression polymorphism of *Sm*PoMucs remained unclear. Our recent finding that Trichostatin A, a modifier of chromatin structure, influences *Sm*PoMuc transcription patterns [13] suggests that epigenetic mechanisms participate in transcription control.

Epigenetic information is information on the status of gene activity that is heritable, for which changes are reversible and that is not based on the DNA sequence [14], [15], [16]. The scientific debate about the reason of the evolution of an epigenetic inheritance system (EIS) in most organisms is intense. Others and we have suggested that EIS provides a basis for modifications in the reaction norms that do not require changes of genotypes [17], [13], resulting in increased phenotypic plasticity at the individual level or increased phenotypic variability at the population level. If EIS influences the capacity to generate different phenotypes, both the better adapted phenotype and the capacity to generate this phenotype will be selected for and carried into the next generation. This hypothesis has been largely validated in the malaria parasite *Plasmodium falciparum* which displays “Clonally Variant Gene Expression” (CVGE) [18]. Genes that show CVGE are present in multicopy, such that individual parasites within an isogenic population express these genes at very different levels, often fully active or completely silenced. Their transcriptional patterns are clonally transmitted to the next generations through asexual multiplication, and stochastic changes of the transcription level occur at low frequency. This bet hedging strategy allows for stochastic generation of phenotypic diversity and can be controlled by epigenetic based events, similar to those described for the *var* gene family. The *var* genes encode the red blood cell surface antigen *P. falciparum* erythrocyte membrane protein 1 (PfEMP-1) and their “CVGE” regulation strategy is responsible for surface antigen variation that ultimately results in immune evasion. In this context, the EIS that leads to “CVGE” allows for rapid adaptation to the ever-changing vertebrate immune environment. In *S. mansoni* miracidia, we have shown that epigenetic-based events influence the phenotypic plasticity in populations [13] and particularly regulate *Sm*PoMuc gene expression. To gain further insight into the precise mechanism of regulation of these genes, in the present study we investigated the genetic and epigenetic changes that occurred during the evolution of the phenotypic compatibility polymorphism in two *S. mansoni* strains. We focused on the sequences of the promoters of active *Sm*PoMuc genes and investigated whether there exist differences in the promoter sequences between *S. mansoni* compatible and incompatible strains. Our study reveals that IC and C strains display very little within strain genetic variability, and limited nucleotide differences between promoter sequences of the two strains, but show strong chromatin structure differences. These chromatin structures are heritable throughout the life cycle and transmitted to the next generation, therefore demonstrating that EIS can control a heritable adaptive trait, such as compatibility polymorphism.

## Results

### Transcription of *Sm*PoMuc genes is different in IC and C strains of *S. mansoni*

**Figure 1:**
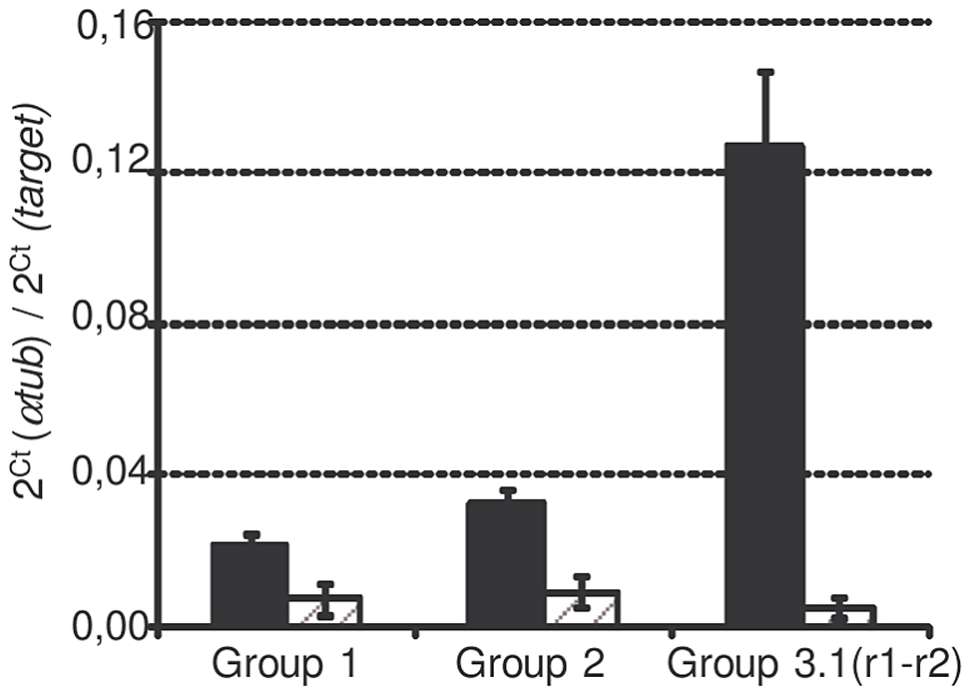
*Sm*PoMuc expression of each group in C and IC strains. mRNA were extracted from miracidia pool from the IC (black bars) and C (dashed grey bars) strains and qPCR were performed with primers targeting *Sm*PoMuc group 1, group 2, group 3.1(r1–r2). Results represents the mean value of 3 biological repeats, * indicates a p-value below 0.05.

*Sm*PoMuc genes are classified into 4 groups (Roger et al. 2008) according to their 3′region: group 1 to 4. Group 3 is itself divided into subgroups (3.1, 3.2, 3.3 and 3.4). *Sm*PoMucs genes have a 5′ region containing a variable number of tandem repeats (exon2), which have been previously called r1 and r2 [8]. r2 exclusively occurs in the group1 and 2 and the intermingled r1–r2 exclusively occurs in the subgroup 3.1, which is present in several copies with either the r1–r2 intermingled repeats or with r1. Due to the very high degree of sequence similarity between the *Sm*PoMuc groups, specific transcriptional analyses of the different *Sm*PoMuc groups were only possible for groups 1, 2 and 3.1(r1–r2). The transcription levels of these groups were compared between miracidia of the IC and C strains. *Sm*PoMuc gene groups 1, 2 and 3.1(r1–r2) are 2.2 to 4.9, 2.5 to 6.7 and 18.6 to 59.7 fold more transcribed in the IC than in the C strain, respectively (fig. 1). The 3.1 subgroup containing intermingled r1–r2 repeats is highly transcribed in the IC strain but was practically undetectable in the C strain. This result is consistent with a previous study on individuals of the IC and C strains, which showed that variants containing the r1–r2 combinations are only expressed in the IC strain [8].

### The *Sm*PoMuc minimal promoter region is located within 1,000 bp upstream of the TSS

**Figure 2:**
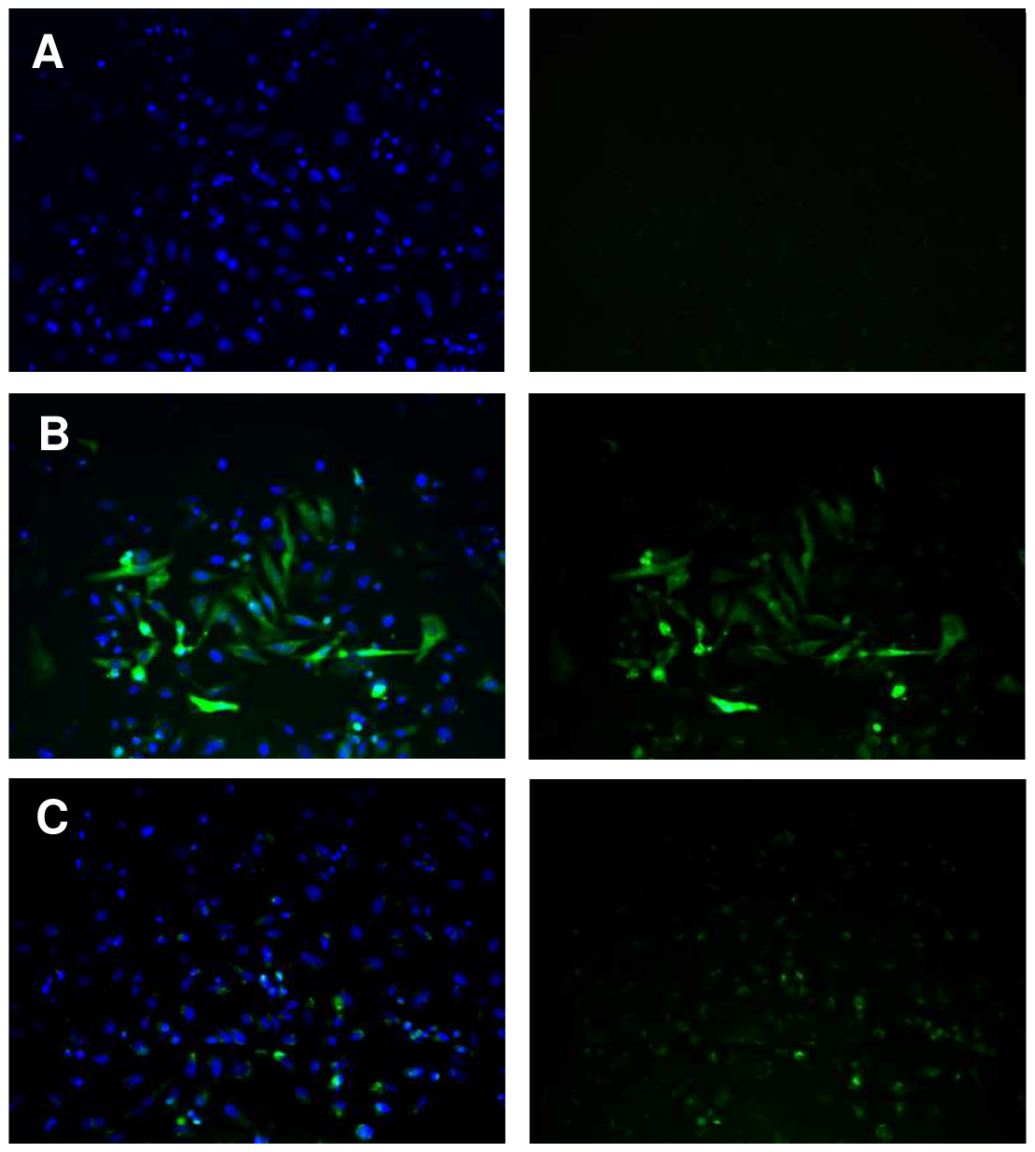
Expression of EGFP under control of the *Sm*PoMuc group 3.1(r1–r2) promoter in HeLa cells. HeLa cells were transiently transfected with (A.) promoterless pEGFP1 vector backbone, (B.) pCMV-EGFP or (C) pSmPoMuc-EGFP. Cell nuclei were labelled with DAPI. Visualization of the fluorescence separately (right panel) or overlaid (left panel) is presented. Magnification is ×400.

To investigate the mechanisms underlying differences of transcription between *Sm*PoMuc groups and subgroups, we characterized the minimal promoter region of the *Sm*PoMuc genes. We sequenced a region spanning 1.04 to 2.00 kb upstream of the transcriptional start site (TSS) for 4 groups of *Sm*PoMuc (Groups 1, 2, 3.1 and 3.1(r1–r2). We produced a PCR product of a 996 bp of the region of the promoter of the group 3.1(r1–r2) and a PCR product of 1002 bp of the group 3.1 just upstream of the transcriptional start site. Plasmids containing these sequences upstream of a reporter gene (EGFP) were transfected into HeLa cells and fluorescence was observed under a microscope (fig. 2). These experiments showed that these sequences are sufficient to drive the heterologous expression of the reporter gene and contain the minimal promoter sufficient for transcription.

### Sequence variations of promoter regions of *Sm*PoMuc genes between *S. mansoni* IC and C strains are small

**Figure 3:**
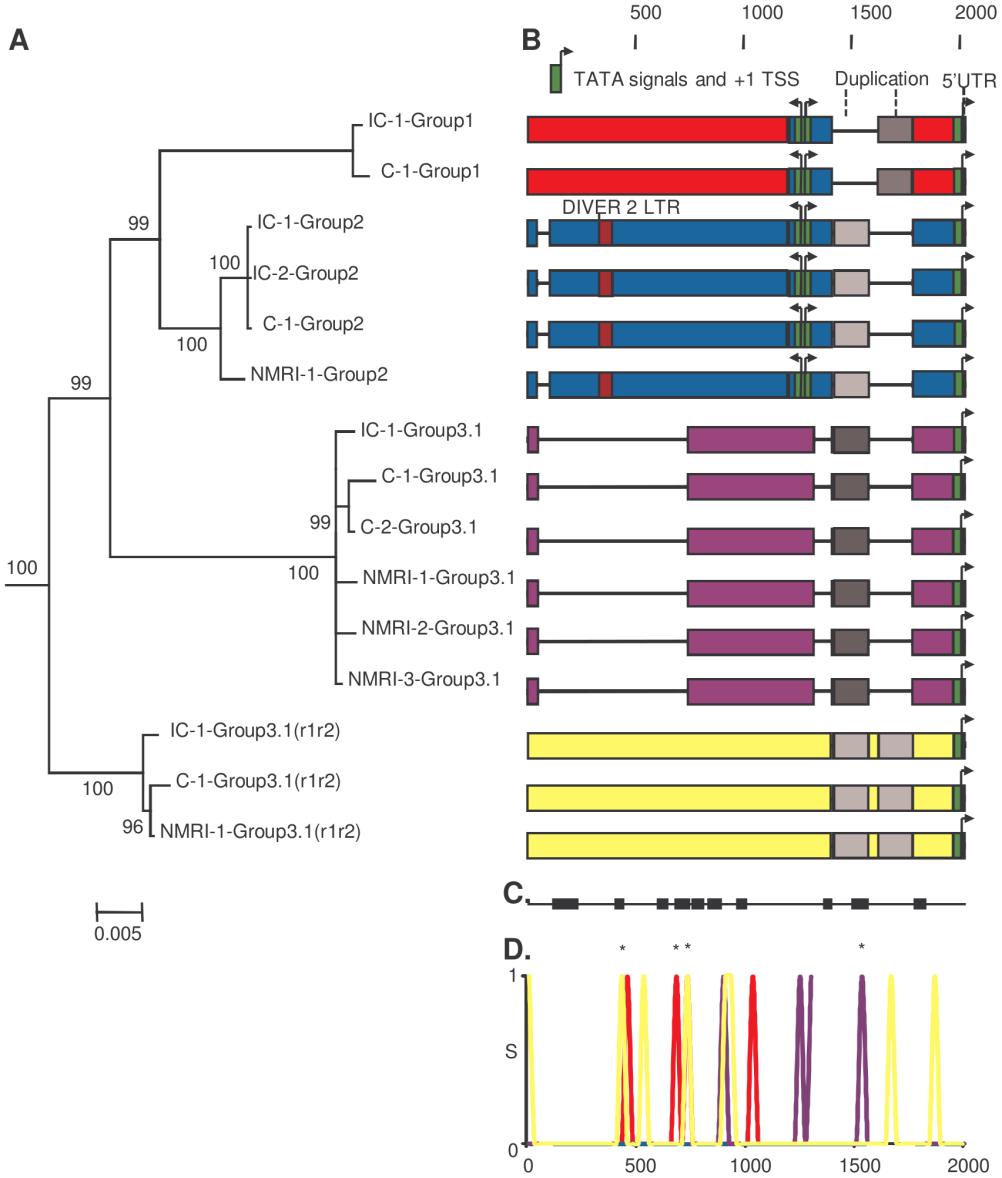
Paralogous and orthologous relationships among *Sm*PoMuc promoters between *S.* *mansoni* IC and C strains. (A.) Bayesian analysis of phylogenetic relationships among *Sm*PoMuc promoter sequences with posterior probability values above 70 indicated on associated nodes. (B.) Schematic diagram of aligned *Sm*PoMuc promoter sequences corresponding to sequences in panel A. Numbers show the nucleotide position in relation to the transcriptional start site in the alignment. We annotated the sequences by colour-coding blocks of less than 95% identity: Group 1 (red), Group 2 (blue), Group 3.1 (purple) and Group 3.1(r1–r2) (yellow). The 5′UTR were characterised and are represented in orange. TATA signals, here in green, and Transcription Starting Sites (+1 TSS) were predicted using Neural Network Promoter Prediction Tool. Deletions are represented by black lines. A recombination event was detected from Group 2 to Group 1 promoter sequences (in blue in Group 1 sequences). One duplication event resulted in an insertion in Group 3.1(r1–r2) (in greys). Traces of a retrotransposon insertion (DIVER2 – LTR) are present in Group 2 (in burgundy). (C.) Black blocks are the conserved regions among groups of *Sm*PoMuc promoters, ignoring the variation within groups of promoters (nucleotide positions as above). (D.) Number of substitutions per site among promoter sequences between strains within *Sm*PoMuc groups (S on Y-axis, colour-codes described above), along the sequence alignment (nucleotide positions on X-axis as above). * are the substitutions between the two strains positioned within regions conserved among groups of promoters. The number of substitutions among *Sm*PoMuc promoters between the two strains varied from 0 in Group 2 to 8 in Group 3.1(r1–r2). No substitution was observed in TATA signals and TSS sites between the two strains. The sequences have GenBank accession number JQ615951 to JQ615965 (See Table S1 for details).

As a first approach to investigate a putative genetic basis for the difference in transcription levels between strains, we investigated the paralogous and orthologous relationships between the four groups of *Sm*PoMuc gene promoters and between the two *S. mansoni* IC and C strains using phylogenetic analysis, reciprocal BLAST dot-plots and comparison of repetitive elements, duplication, recombination events and gene conversions (fig. 3). We annotated the sequences and visualised them by colour-coding of blocks with less than 95% identity (fig. 3). A recombination event was detected using BootScan [19], [20], Maximum Chi Square [21], [22] and Sister Scanning [23] methods in RDP3 and the recombination break points were putatively identified (fig. 3). In both strains we observed one duplication in group 3.1(r1–r2) promoters resulting in an insertion, several insertions/deletions (indels) including one large deletion in group 3.1 promoters and probably a recombination event from the group 2 to group 1 promoter. High similarity to a repeated DNA element was detected in the group 2 promoter; however, it constituted only a small fragment of the complete repeat – 61 bp out of 385 bp of the DIVER2 LTR (*Drosophila*).

The estimated divergence time between the IC and C *S. mansoni* strains is about 400 years [6] and the promoter sequences between the two strains are highly conserved (0.000–0.004 net substitutions per site, Table 1). The number of fixed differences between the two strains varied between 0 in the promoter region of *Sm*PoMuc group 2 genes, to 3 in group 3.1, 4 in group 1 and 8 in group 3.1(r1–r2) (Table 1). No substitution was observed in the TATA signal, nor in the TSS regions or in putative regulator binding sites of the promoters between the two strains. *Sm*PoMuc promoter sequences were divided into four paralogous sequence groups and sequence differences between strains (orthologous relationships) within groups were much less than the differences observed between groups of the *Sm*PoMuc gene family - net substitutions per site varied from 0.000–0.004 within groups of promoter sequences between strains compared to 0.024–0.041 between promoter groups (Table 1). The number of *Sm*PoMuc promoter sequence differences between strains was equal to or slightly higher than the number of sequence differences for the promoter of the single copy gene *SmFTZ-F1* [24] which shows no difference between strains (Table 1). Six of 14 microsatellite loci also showed no sequence differences between the two strains (one unique allele). The two strains share the molecular evolution and phylogeny of the promoter region of the four groups of the *Sm*PoMuc gene family (fig. 3) – indels, recombination and duplication events. These findings indicate that the divergence between groups of the *Sm*PoMuc gene family from a common gene ancestor is ancient and largely predates the time of separation between the IC and C strains.

**Table 1:** Sequence variation between *Schistosoma mansoni* IC and C strains and between groups of *Sm*PoMuc promoter sequences.

Sequences	Seq. length (kb)	*N*	Nb polym. sites	Nb mutat.	Nb fixed diff. IC *vs.* C	*D_a_* IC *vs.* C	*D_a_* group 1 *vs.*	*D_a_* group 2 *vs.*	*D_a_* group 3.1 *vs.*	*D_a_* group 3.1(r1–r2) *vs.*
*Sm*PoMuc promoters										
Group 1	1.80	2	4	4	4	0.002	-	0.021	0.031	0.035
Group 2	1.74	3	0	0	0	0.000		-	0.040	0.026
Group 3.1	1.04	3	4	4	3	0.003			-	0.040
Group 3.1(r1–r2)	2.00	2	8	8	8	0.004				-
*Sm*PoMuc introns										
Group 1	7.14	2	12	16	16	0.002	-	0.068	0.031	
Group 2	7.72	2	32	32	32	0.004		-	0.068	
Group 3.1	7.11	2	43	43	43	0.006			-	
*SmFTZ-F1* promoter										
*SmFTZ-F1*	1.4	3	0	0	0	0.000				

Seq. Length, sequence length; *N*, number of sequences; Nb polym. sites and Nb mutat., number of polymorphic sites and mutations, respectively; Nb fixed diff. IC *vs.* C, number of nucleotide differences between IC and C strains' promoter sequences; *D_a_* IC *vs.* C, number of net nucleotide substitutions per site between IC and C strains' promoter sequences; *D_a_* group X *vs.*, number of net nucleotide substitutions per site between groups of promoter sequences.

### Low level of promoter nucleotide diversity within the IC and C strains

At this stage of the study we hypothesized that *Sm*PoMuc expression differences in C and IC strains could be due to nucleotide differences in the promoter regions of the genes. The sequencing of 1.4 kb of *Sm*PoMuc group 1 promoter region for 20 and 18 individuals of the IC and C strains respectively, revealed a very low number of alleles and genotypes (Table 2) – one genotype in the IC strain and 3 genotypes in the C strain. In the C strain, sequence variation was minimal, with the three alleles differing by only one base pair from each other, resulting in insignificant nucleotide diversity (Table 2). All individuals were homozygotes. The IC strain allele of the *Sm*PoMuc promoter group 1 differed from the three C strain alleles by four to five base pairs, a sequence divergence of 0.29 to 0.36%. In summary, nucleotide sequence differences between the two strains are surprisingly small.

**Table 2:** Sequence diversity of the promoter of *Sm*PoMuc group 1 genes for *Schistosoma mansoni* IC and C strains.

	*N*	*A*	*S*	Gen. Div. ± SD	Nuc. Div. ± SD	Tajima's *D*	*P*
IC	20	1	0	0.00±0.00	0.000±0.000	0.000	1.000
C	18	3	2	0.22±0.12	0.000±0.000	−1.508	0.048

*N*, number of *S. mansoni* samples; *A*, number of alleles; *S*, number of substitutions; Gen. Div. ± SD and Nuc. div. ± SD, expected unbiased gene diversities and nucleotide diversities, respectively. Tajima's *D*, *P*, Tajima's *D* and test of significance of *D*, respectively.

Sequence length: 1.4 kb.

### *Sm*PoMuc group 1 promoter population sequence difference between IC and C strains is not higher than the average genome-wide difference

Promoter diversity within strain and divergence between strains of *Sm*PoMuc group 1 genes were similar to those of 14 microsatellite loci that can be used to reflect genome-wide diversity and divergence [25]. The promoter diversity of *Sm*PoMuc group 1 was 0.00 (one allele) in the IC strain compared to 0.22 (3 alleles) in the C strain (Table 2), while expected heterozygosity was 0.000 (one allele) for both strains for 14 microsatellite loci (Data not shown). All individuals were homozygotes. Six out of 14 microsatellite loci showed no divergence between the two strains. At eight microsatellite loci, the IC strain alleles differed from the C strain alleles by one to eleven microsatellite repeats. The promoter region of the single copy *Sm*FTZ-F1 gene displayed a unique sequence common to the two strains. We estimated extremely high and significant genetic differentiation between the two strains for both *Sm*PoMuc group 1 promoter sequences and microsatellite loci using *θ*, *Φ_ST_* and *R_ST_* estimators (Table 3). However, we detected almost no heterozygotes and highly significant inbreeding coefficients *f* in both strains and for both *Sm*PoMuc group 1 promoter sequences and the microsatellite loci (Table 3). Therefore the high values of divergence are likely the result of the bottleneck induced during the care of the life cycle in the laboratory in the two strains as discussed previously [25]. Nonetheless, the distribution of alleles matched the pattern of differentiation as we detected fixed alleles that were different in the two strains. We reasoned that the small genetic differences in the promoter region are simply a by-product of clonality and not the reason for expression differences. We therefore explored an alternative hypothesis, *i.e.* that the expression differences are due to dissimilarity in the epigenetic information.

**Table 3:** Genetic differentiation between *S. mansoni* IC and C strains estimated by *Φ_ST_* and *θ*, and inbreeding coefficients *f* within strains.

	*Φ_ST_*/*R_ST_*	*θ*	*f* in IC	*f* in C
*Sm*PoMuc promoter group 1	0.974^S^	0.898^S^	N/A	1.00^S^
Microsatellites	six loci N/A (one allele)	six loci N/A (one allele)	fourteen loci N/A	N/A
	eight loci 1.000^S^	eight loci 1.000^S^		

S : significant departure from 0 at *P* = 0.05.

### HDAC inhibitors have an effect on *Sm*PoMuc transcription

As the difference in *Sm*PoMuc transcription phenotype cannot easily be explained by genetic differences in the promoter region, we investigated the putative implication of epigenetic mechanisms. As a previous study had shown that histone modifications are clearly involved in *S. mansoni* epigenetic mechanisms [13], [26], we tried to influence the epigenotype and phenotype (*Sm*PoMuc expression pattern) of *S. mansoni* using trichostatin-A (TSA) that is a specific and reversible inhibitor of class I and II histone deacetylases (HDAC). Treatment with this drug prevents histone deacetylation and is expected to increase the overall acetylation of histones and therefore gene expression [26] [27]. The influence of TSA treatment on the transcription of *Sm*PoMuc genes (group 1, 2 and 3.1(r1–r2) of both C and IC strains was tested in miracidia larvae exposed during 4 h to the drug. A Friedman non-parametric test was performed to test the significance of the TSA effect (Figure S1). We observed a statistically significant increase in transcription of groups 1 and 2 after TSA treatment in the IC strain only (p-value = 0.05). This indicates that changes in histone acetylation correlate with increased expression for *Sm*PoMuc group 1 and 2 in the IC strain and has no effect in the C strain. Control genes were also tested for their response to TSA in order to determine that its effect was not pleiotropic. No effect of TSA was observed for these genes (GAPDH, Smp_011030, Smp_152710.1, Smp_054160, Smp_158110.1, GST.B, Glyaxalase, data not shown).

### Strain hybrids express both C and IC strain specific *Sm*PoMucs

**Figure 4:**
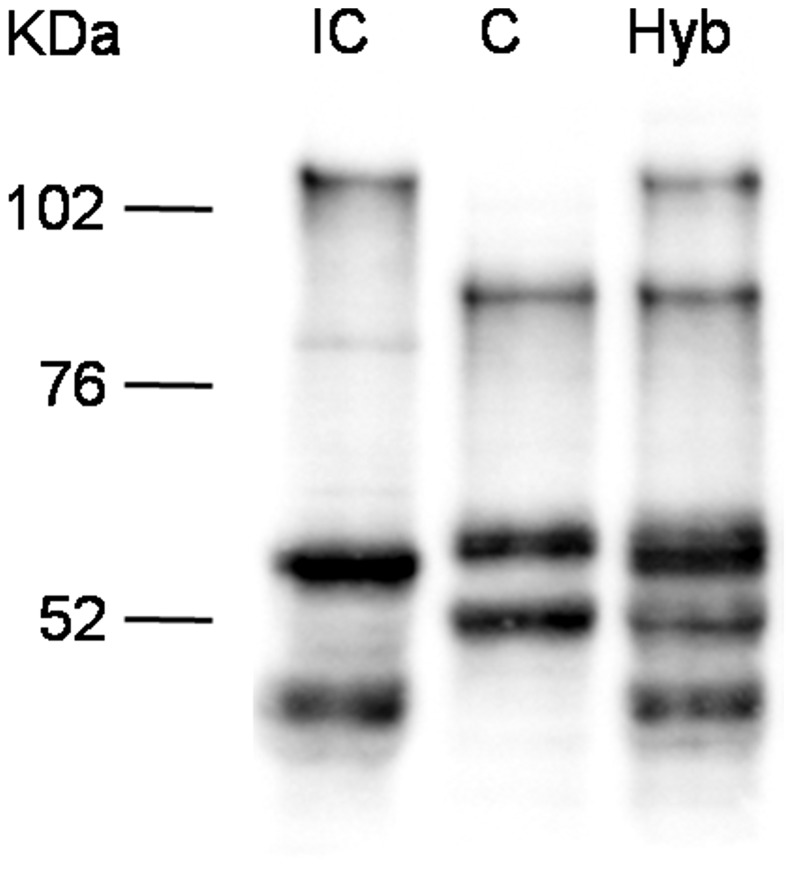
Western blots analysis on *Sm*PoMuc proteins from C/IC hybrids and C and IC control strains. Western blot experiments were performed on miracidial protein extracts from C/IC hybrids and C and IC control parental strains.

Since the TSA treatment influences overall histone acetylation, it could not be excluded that the observed effect is an indirect one and that *Sm*PoMuc expression control is posttranscriptional and/or posttranslational such as selective RNA or protein degradation. We reasoned that in the offspring of crosses between the IC and C strains transcriptional control would produce an additive pattern of *Sm*PoMuc proteins, while control by selective degradation of gene products would produce a subtractive pattern. Western blots show that in miracidia that are produced from crosses between the strains an additive pattern of the C and IC specific bands can be observed (fig. 4). This indicates that regulation operates at the transcriptional and not the post-transcriptional level and further supports the view that chromatin structure plays a role in the generation of specific *Sm*PoMuc profiles for each strain.

### There are strong chromatin status differences in the *Sm*PoMuc promoters between *S. mansoni* C and IC strains

**Figure 5:**
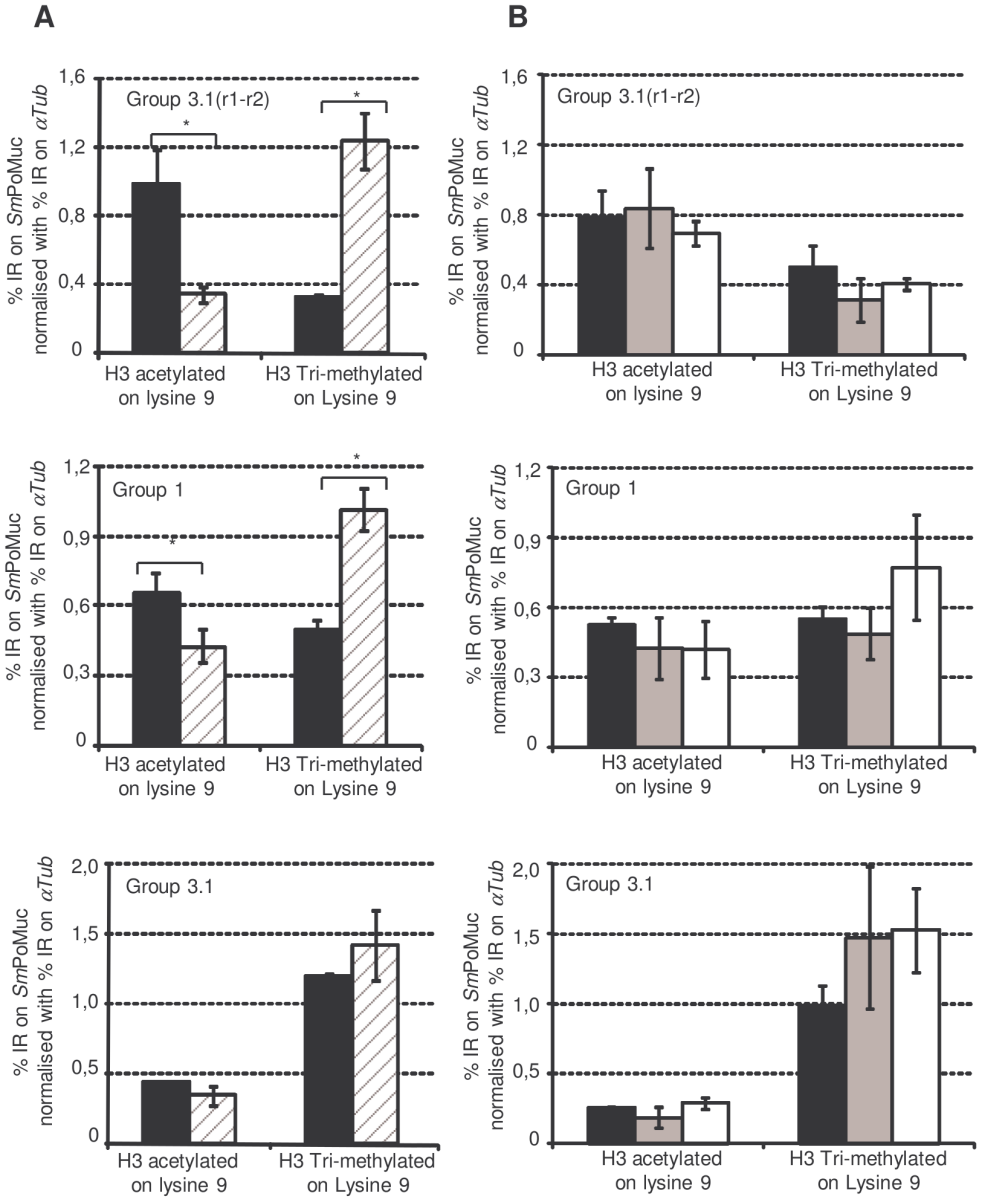
Chromatin immunoprecipitation on *Sm*PoMuc promoter regions. Experiments were performed on chromatin isolated from miracidia from both the IC (black bars) and C strains (dashed grey bars) (A.) and on chromatin isolated from IC strain miracidia (Black bars), cercaria (grey bars) and adults (white bars) (B.). ChIP was performed with antibodies against H3 acetylated on lysine 9 and H3 tri-methylated on lysine 9. Immunoprecipitated chromatin was analysed by qPCR using primers that hybridize with specific sequences of *Sm*PoMuc group 3.1(r1–r2), group 1and group 3.1. Results represent the percentage input recovery (%IR) on target genes normalised with %IR of a reference gene (αTub). Results are the average of 3 biological samples. * indicates a p-value<0.05 of a student t test.

Since all our experiments had delivered results in favour of a difference in chromatin structure of the *Sm*PoMuc locus between strains, we decided to investigate the chromatin status in these loci. The occurrence of DNA methylation in *S.mansoni* is currently debated [28] [29]. To test for DNA methylation in the promoter region of *Sm*PoMucs we performed bisulfite genomic sequencing of DNA from miracidia using *in-vitro* methylated DNA as a positive control. We did not detect any methylated cytosine in the target region while 98% of the CpGs of in-vitro methylated DNA scored methylation positive. Our results are in line with earlier results showing that DNA methylation is rare from genes in *S.mansoni* [29] [28]. We then performed Chromatin ImmunoPrecipitation (ChIP) experiments to check for histone modifications in the promoter regions. Due to the high similarity between the different groups of *Sm*PoMuc promoters, ChIP-qPCR (quantitative Polymerase Chain Reaction) analysis was possible only in degenerate regions. Therefore, the chromatin structure analysis was performed on the promoter regions of *Sm*PoMuc groups 1, 3.1 and 3.1(r1–r2). ChIP experiments were performed using an antibody that recognised Histone 3 acetylated on lysine 9 (H3K9Ac) and Histone 3 tri-methylated on lysine 4 (H3K4Met3) which are euchromatic marks and an antibody that recognised H3 tri-methylated on lysine 9 (H3K9Met3), which is a heterochromatic mark. Immunoprecipitation with the antibody that targets H3K4Met3 did not show any enrichment in the *Sm*PoMuc region tested for either the IC or C strains whereas controls, αTub (Smp_090120.2) and 28S (Z46503.1) were positive (data not shown). The H3K4Met3 mark is usually very sharp and difficult to localise by target approach.. Both *Sm*PoMuc group 1 and 3.1(r1–r2) from the IC strain displayed a higher level of H3K9Ac compared to the C strain (fig. 5). Consistent with this result, the C strain displayed a higher level of the heterochromatic mark (H3K9Met3) for group 1 and 3.1(r1–r2). These results have been obtained with several generations of the parasite, demonstrating that the phenotype is transmitted to the next generation.

In the IC strain, epigenetic marks showed differences among *Sm*PoMuc groups 1, 3.1 and 3.1(r1–r2) (Figure S2). The promoter of group 3.1(r1–r2) is the most acetylated and the least heterochromatic. This result is consistent with expression analysis after TSA treatment where no effect of TSA was observed for the expression of group 3.1(r1–r2). This absence of an effect of TSA may be explained by the fact that acetylation on this promoter is already saturated and cannot be further increased as previously observed for H4 acetylation in the promoter region of HDAC1 in *S. Mansoni* [26].

The chromatin status in the promoter sequence of *Sm*PoMuc groups 1, 3.1 and 3.1(r1–r2) was also investigated in the IC strain in cercaria and adults where *Sm*PoMuc genes are not expressed. The level of the heterochromatic and euchromatic marks was the same as in miracidia and this level was maintained through several generations (Figure S3).

## Discussion

The host-parasite arms race determines that variability-generating processes are crucial for survival on both sides of the interaction (red queen hypothesis, [2]). The mechanisms that are responsible for these (heritable) phenotypic variations are a current and fundamental question in evolutionary biology. Traditionally, random genetic changes are seen as the sole source of phenotypic variation. But the picture is probably more complex: heritable adaptive phenotypic shifts could be partly controlled by epigenetic factors that were underrated until recently [30], [31]. A high rate of heritable epigenetic changes would generate phenotypic variation, which in turn could allow a rapid response to selection pressures [13]; [32]. This could allow for a transient and efficient response to changes in the environment, and could subsequently be followed by stabilization through genetic changes [33], [34]. Epigenetic modifications affect the transcription status of a gene in a heritable way without changes in the DNA sequence [14], [15], [16] and epigenetic information can be based on a chromatin marking system. Chromatin exists either as a relaxed structure that is permissive to gene expression and is called euchromatin, or as a condensed structure that is typically silent and is called heterochromatin [35]. Therefore, these different chromatin states alter gene expression and, ultimately, influence phenotypic outcomes without changes to the DNA sequence. The evolutionary implications of epigenetic inheritance systems and their potential link to stress-induced phenotypic variation have been discussed in several models [36], [37], [38], [31], [39], [40], [41] as well as in the specific context of host-pathogen interaction [42].

While it is clear now that induced epigenetic modifications are heritable [43], there are very few reports that show that epigenetic events lead to modification of gene expression profiles, production of new phenotypes and adaptation to the environment [44].In the present work, we addressed the question of the relative importance of genetic and epigenetic differences between two strains of *S. mansoni* that show clear differences in an ecological important adaptive trait: the capacity to infect their intermediate host. We had previously identified the *Sm*PoMuc genes as surface molecular markers important for host compatibility. These markers encode mucins that display an extraordinary level of polymorphism, although they are produced from a relatively small number of very similar genes.As we had shown that nucleotide differences in the coding region could not explain differences in transcription, we focused therefore on the promoter regions in the present work. Our comparative survey of sequence variation in the different groups of *Sm*PoMuc gene family from IC and C strains revealed a high level of conservation of the promoter sequences of *Sm*PoMuc genes between the two strains. The molecular evolution of *Sm*PoMuc promoters was uniform between all strains analysed, IC, C and NMRI. The sequence differences between the IC, C and NMRI strains within each group of *Sm*PoMuc promoter were small, and the number of substitutions between the IC and C strains was equal or slightly higher than in the monomorphic single-copy gene *SmFTZ-*F1 and consistent with sequence differences at 14 microsatellite loci. To assess whether substitutions between the two strains could have an effect on transcription, we searched for functional regions of the active promoters. None of the substitutions between the IC and C strains occurred in the TATA signal, putative transcription factor binding sites or TSS regions. The nucleotide differences between the two strains consisted of zero in group 2 to eight substitutions in group 3.1(r1–r2), resulting in net nucleotide substitutions per site similar or lower than the ones observed in presumably neutral *Sm*PoMuc introns (Table 2). At the population level, our analysis of *Sm*PoMuc group 1 promoters in the IC and C strains revealed very low allelic and nucleotide variability within strain and high allele frequency differences between the IC and C strains due to fixed substitutions. All individuals were homozygotes at *Sm*PoMuc group 1 promoter, similarly to the genotypes at 14 microsatellite loci, suggesting that *S. mansoni* strains present genome-wide homozygosity. Both strains are characterised by a high significant inbreeding coefficient, resulting from high clonality in the two strains [25], which may have arisen because of the bottleneck due to the strain maintenance in laboratory conditions. Despite the lack of diversity within strains, alleles fixed in each strain for the *Sm*PoMuc group 1 promoter and nine microsatellites were different, resulting in high genetic differentiation between the two strains as estimated by *F_ST_*. This contrasted with the promoter of the single-copy gene *Sm*FTZ-F1 and six microsatellite loci, which displayed a unique sequence common to the two strains.

In summary, our analysis of the genetic information shows that (i) both strains are genetically monomorphic, including the *Sm*PoMuc promoter regions, (ii) both strains are different in terms of alleles, i.e. they do not share the same alleles, but (iii) these alleles are similar or display low number of base substitutions (outside functional regions). It could be argued that the small nucleotide differences observed between the two strains are sufficient to provoke modulation of histone modification. Such a leverage effect of SNPs cannot be excluded but has so far not been observed in heavily studied models such as human, *Drosophila melanogaster* and *Arabidopsis thaliana*. It could also be the case that strain-specific loci exist that regulate the chromatin structure of the *Sm*PoMuc genes *in trans* or *in cis* (upstream of the minimal functional promoter). However, previous work has compared the proteomes of both C and IC strains [7] and did not pinpoint any major regulators that may be responsible for such a phenotype. In view of these results, we argue that genetic differences between sequences within each group of *Sm*PoMuc promoters were unlikely to solely dictate the high level of variation in *Sm*PoMuc transcription and compatibility polymorphism phenotypes.

We therefore further investigated the epigenetic basis for such phenotypes. TSA treatment was used to study the impact of overall acetylation status of histones on miracidia larvae where *Sm*PoMuc is expressed. This drug is known to be a specific histone deacetylase (HDAC) inhibitor and has been previously shown to influence phenotypic traits in *S. mansoni* [13]. A dose dependant effect of TSA was observed for *Sm*PoMuc expression (all groups taken together) in the IC strain whereas no effect was observed in the C strain. This result suggests that the acetylation status of histones in the promoter sequences is differentially regulated between the IC and C strains. HDACs seem to play a more prominent role in regulating the acetylation level in the IC strain that allowed us to pinpoint a TSA effect in this strain. More specifically, we report a TSA effect on groups 1 and 2 of the IC strain whereas no effect is observed for group 3.1(r1–r2) for which acetylation is the strongest. This also suggests that a differential regulation by HDAC exists between the *Sm*PoMuc groups in the same strain. Further support for regulation on transcriptional level comes from a crossing experiment in which strain hybrids were produced. Western blots show that in the hybrids, both the C-specific and the IC-specific *Sm*PoMucs are expressed. One could hypothesize that production of *Sm*PoMuc variants is due to post-transcriptional strain-specific regulation. In this scenario all genes would be expressed, but the gene products would be processed in a strain-specific form. In the hybrids, in which the hypothetical post-transcriptional regulation pathway for both strains is present, we should have seen a diminution of non-IC and the non-C *Sm*PoMuc forms. This was not the case. In summary, all lines of evidence point towards a chromatin-based regulation of *Sm*PoMuc expression.

The chromatin configuration was further investigated by ChIP analysis using antibody that recognises heterochromatic and euchromatic marks. ChIP results clearly demonstrate that different epigenetic marks occur on the *Sm*PoMuc promoter of group 1 and group 3.1(r1–r2) between the IC and C strains likely resulting in a different chromatin configuration. On these loci, chromatin is indeed more enriched in H3 acetylated on lysine 9 in the IC compared to the C strain and less enriched in the opposite mark, H3 trimethylated on lysine 9. Therefore, the local chromatin structures differ between the two strains for groups 1 and 3.1(r1–r2) and are consistent with expression data as stronger acetylation correlates with enhanced expression. Importantly, H3K9Met3 and H3K9Ac marks are maintained through the cercarial and adult stages at which the genes are not expressed. This persistence of the chromatin mark throughout other stages of the *S. mansoni* life cycle is a crucial result as this is a necessary condition for the epigenetic mechanism to act as a heritable trait. Similarly, several CVGE genes of *P. falciparum* that display a bistable chromatin state to regulate their expression in the intraerythrocytic stages have been shown to maintain their epigenetic marks during trophozoite and schizont stages, the other asexual stages at which these genes are not expressed [45].

It is now established that the phenotype is not onlya product of genetic processes, but expression of an ensemble that is composed of genetic and epigenetic components. Others and we have proposed that this additional system allows for rapid adaptive evolution without necessarily changing the genotype initially. A theoretical framework for this model was provided by Pal and Miklos (1999) [17], and more recently by Klironomos, Berg and Collins (personal communication). Essentially, these authors propose that a higher rate of random changes in epigenetic marks compared to genetic mutations transmitted from one generation to the next in a population generates increased phenotypic variations that can be selected for if the environment changes. In this sense, epigenetic modifications provide a source of rapid and reversible phenotypic variation and are therefore expected to be major players in the context of host-pathogen interaction where selection pressures are strong and evolution is fast [42], [18]. In this context, epigenetic based events to generate variability of surface antigens of parasites perfectly matched to this theory. For exemple, VSP diversification of *Giardia sp.* likely occurs by epigenetic mechanisms involving the histone acetylation status [46] and/or RNAi [47]. Chromatin remodeling proteins and histone modifications have been shown to play a role in VSG expression site silencing [48] and *Plasmodium* Var diversification is orchestrated by multiple epigenetic factors including monoallelic transcription at separate spatial domains at the nuclear periphery, differential histone marks on otherwise identical *var* genes, and *var* silencing mediated by telomeric heterochromatin [49]. On the host side, genetic and epigenetic crosstalks have been previously demonstrated in the generation of a high level of polymorphism of the receptors of the adaptative immune system [50], [51]. Therefore, all these variability generating mechanisms are examples of local adaptation to an ever-changing environment where epigenetic based events are used to rapidly produce new phenotypes and potentially induce rapid evolutionary change of genes that are under pressure. In our work, we show that two population of *S. mansoni* with distinct phenotypic traits, in particular their compatibility with a reference host, show low nucleotide differences in both coding sequence and promoters of *Sm*PoMuc but high epigenetic differences in the promoter regions. Both parasite populations are in a situation where the fitness value of genetically encoded phenotypes has not changed significantly, but epigenetic variations have produced phenotypic variants that are adapted to different environments (compatible hosts).

While we have compared only South American strains, our observations suggest a scenario for the adaptation of *S. mansoni* to the new world host: in the 15th–16th century the ancestral strain of contemporary strains IC and C migrated via the slave trade from Africa to the West Indian Islands and the South American continent, respectively [6]. There, they had to adapt to a new intermediate host. The initial bottleneck resulting from the migration of only a limited number of parasites and the expected strong selective pressure acting on both genetic and epigenetic variants of the key-molecules for compatibility with the new snail hosts, *Sm*PoMucs, may have significantly reduced genetic and epigenetic variation in the newly formed laboratory IC and C strains compared to the ancestral strain. Now, it is likely that epigenetic variation retained from the ancestral strain and the higher rate of occurrence of epigenetic changes in subsequent generations, rather than the strain genetic variation, enabled the parasite to adapt rapidly to their host and new environment. A conundrum with the “epigenetic mutation system first” hypothesis is that epigenetic information concerns the transcriptional activity of a gene but not its coding potential, in other words, a gene can be switched on and off by the surrounding chromatin but the resulting protein cannot be changed. Loss of function of genes can easily be imagined through an epigenetic mechanism, but for gain of function a complex inhibitor-based mechanism would be necessary. The classical Ohno hypothesis of gene duplications as way to provide material for evolution [52] could deliver a solution. Rodin and Riggs have shown that duplicated genes have a tendency to be heterochromatic [53]. It is interesting to note that the *Sm*PoMuc proteins, essential for host compatibility, are encoded by duplicated genes. Our analysis shows that the duplication events predate the IC/C separation and occurred in the strain's common ancestor, *i.e.* gene duplication was not a result of divergence of the two strains. We postulate that *Sm*PoMuc duplicated genes provide an additional system for phenotypic variation. Duplicated genes are randomly modulated in their relative transcriptional activity through chromatin structure changes as evidenced by our current and previous results [13], resulting in new combinations of expressed *Sm*PoMuc genes and subsequent increased phenotypic variation. If the parasite encounters new intermediate hosts, the probability for the phenotypes to match is increased, thus allowing for adaptive evolution.

Therefore, our work shows that in a gene family that codes for an adaptive phenotypic trait, epigenetic changes are more important than genetic changes. This finding provides support for theoretical models of adaptive evolution in which epimutations occur more rapidly than mutations.

## Materials and Methods

### Ethics statement

The French Ministère de l'Agriculture et de la Pêche and French Ministère de l'Education Nationale de la Recherche et de la Technologie provided permit A 66040 to our laboratory for experiments on animals and certificate for animal experimentation (authorization 007083, decree 87–848) for the experimenters. Housing, breeding and animal care followed the national ethical requirements.

### Culture of *Schistosoma mansoni*

A compatible strain (C) (Brazilian strain), an incompatible *S. mansoni* strain (IC) (Guadeloupean strain), the reference NMRI *S. mansoni* strain (Puerto Rican strain) and a reference mollusc strain (*B. glabrata* BRE isolated from Brazil) were used in this study. For initial breeding, each strain was maintained in its sympatric (compatible) *B. glabrata* strain, and in hamsters (*Mesocricetus auratus*) as described previously [54]. Adult worms and miracidia were obtained as described previously [8].

### Generation of strain hybrids and Western blot

Individual *B. glabrata* snails were infested with a single miracidium to obtain cercarial clonal populations. Subsequently the sex of the cercariae was determined as described previously [55]. Strain hybrids of *S. mansoni* were produced by infection of mice or hamster with 300 cercariae: 200 males from a clonal cercarial population combined with 100 females from another clonal cercarial population. Different combinations of parental cercariae of the IC and C strains were used, thus generating worm couples in which the male is C and the female is IC or *vice versa*. Eggs were recovered from infected (3 to 6) mice (*Mus musculus*) 12 weeks post-infection. Livers were collected and homogenized, and eggs were filtered and washed. Miracidia were allowed to hatch in spring water and were concentrated by sedimentation on ice for 15 minutes.

1000 Miracidia were incubated in 350 µl UTCD buffer (ultrapure urea 8 M, Tris 40 mM, DTT 65 mM, CHAPS 4%), two hours at room temperature. The extract was cleared by centrifugation for 30 minutes at 1500 g, and the supernatant was collected. Total proteins (5 µg per sample) were separated by 10% SDS-PAGE gel electrophoresis before being blotted on a nitrocellulose membrane (Trans-Blot turbo, Bio-Rad). The membrane was blocked with 5% non-fat dry milk in TBST (TBS buffer containing 0.05% tween 20) one hour at room temperature, and incubated with the primary antibody “anti-*Sm*PoMuc” diluted 1/500 in TBST for 90 minutes at room temperature. This rabbit polyclonal antibody was produced according to standard procedures and was shown to recognise all the *Sm*PoMuc groups [9]. Then, the membrane was incubated with secondary antibody (peroxidase conjugated, purified anti-rabbit IgG) diluted 1/5000 in TBST for 1 hour. After washing 3 times for 10 minutes in TBST, the detection was carried out using the ECL reagents and the ChemiDoc MP Imaging system – BioRad).

### PCR screening for promoters of *Sm*PoMuc genes, cloning and sequencing

We searched for sequences of promoter regions of *Sm*PoMuc genes in the genomic database of the *S. mansoni* NMRI strain (assembly version 3.1) using BLAST searches. Contigs matching to *Sm*PoMuc genes were assembled with the Sequencher software (Gene Codes Corporation) to recover the sequences of the promoter regions of the genes. From the BLAST search and manual assemblage of relevant contigs, scaffolds of promoter regions were constructed for the different *Sm*PoMuc genes in groups 1–4. Primers were designed on these contigs to amplify the promoter regions of the different *Sm*PoMuc genes in the C and IC strains of *S. mansoni*. The DNA templates to generate PCR products were either genomic DNA (C and IC strains), a BAC library (NMRI strain) or a phage library (IC strain). Genomic DNA was extracted from adult worms as described previously [8]. The production of the phage library is described below. Promoter regions were amplified using the Advantage 2 PCR Enzyme System (Clontech) (Table S1 for primer sequences, amplified fragment lengths and sources of DNA). PCR products were either cloned into pCR-XL-TOPO (TOPO TA Cloning kit for sequencing, Invitrogen) and plasmid DNA was purified using the Wizard Plus SV Miniprep DNA purification system (Promega), or sequenced directly. We sent PCR amplificons or plasmids containing the promoter regions to GATC (GATC Biotech, Germany) for cycle sequencing in both directions and performed primer walking up to 2.0 kb upstream of the transcription start sites (TSS) of *Sm*PoMuc genes (for primer sequences see Table S2). We checked trace data and aligned nucleotide sequences manually using the BioEdit software. We scanned the promoter sequences for putative regulator binding sites using the web based interface Program NSITE (Softberry Inc.) (http://linux1.softberry.com/berry.phtml?topic=nsite&group=programs&subgroup=promoter).

### Production and screening of a phage lambda library of IC genomic DNA

The presence of multiple copies of some *Sm*PoMuc genes sometimes prevented the amplification of a single copy and assembly of a gene with its corresponding promoter. To address this problem, we constructed a phage library of the IC strain using the Lambda Fix II vector system from Stratagene. The expected size of inserts was 15 to 23 kb corresponding to the size range of *Sm*PoMuc genes (10–30 kb). Details of the construction of the phage library and screening are available at http://methdb.univ-perp.fr/epievo/. Genome coverage of the library was four fold. The library was screened for *Sm*PoMuc genes using as a probe UR1, a highly conserved intronic sequence spanning the region between two repeat units of the *Sm*PoMuc genes [8]. The probe was labeled with the DIG High Prime DNA Labeling and Detection Starter Kit II using Random primed DNA labeling with digoxigenin-dUTP, alkali-labile and chemiluminescence with CSPD (Roche). Screening was performed according to the manufacturer's instructions. Secondary and tertiary screening rounds were performed with the same probe to isolate individual phage clones. Phages that scored positive for *Sm*PoMuc repeat units were screened by PCR using a combination of diagnostic primers for each group of *Sm*PoMuc genes (Table S2) with the Advantage 2 PCR Enzyme System (Clontech). Selected phages were subsequently purified and used as templates to PCR amplify *Sm*PoMuc group 3.1(r1–r2) as described in the section “PCR screening for promoters of *Sm*PoMuc genes, cloning and sequencing”.

### Sequence variation of promoter regions of *Sm*PoMuc genes between *S. mansoni* IC and C strains

#### Sequence annotation and promoter prediction

The 5′UTR and ORF were previously characterised using 5′RACE-PCR experiments [56]. The core promoter including a TATA box and the TSS was predicted using Neural Network Promoter Prediction Tool (http://www.fruitfly.org/seq_tools/promoter.html) [57]. We identified repetitive elements in the promoter region sequences using the CENSOR software [58]. We searched for duplications, recombinations and gene conversions using dot plots among sequences and the programs RDP3 [20]. *Sm*PoMuc promoter sequences were annotated using CLC Sequence Viewer v6.5.1 (CLC Bio 2011). We colour-coded paralogous sequence blocks, portions of repetitive elements, duplications and recombination to visualise the evolution of paralogous and orthologous *Sm*PoMuc promoter sequences. The number of substitutions per site for pairwise comparisons and searched for conserved regions was calculated with DnaSPv4.50.3 [59].

#### Phylogenetic analysis

We performed Bayesian phylogenetic analyses using MrBayes 3.2.0 [60]. We sampled across the substitution model space in the Bayesian Markov Chain Monte Carlo (MCMC) itself [61]. The model selected was the HKY model. Insertion/deletion (indel) events were coded as binary characters (presence/absence) and included as a separate binary data partition in the analysis [60]. We ran the MCMC for 120,000 generations, trees being sampled every 100 generations. This allowed the final average standard deviations of split frequencies to reach below 0.01 and the potential scale reduction factors (PSRF) for all parameters to be close to 1, indicating that the runs had converged onto the stationary distribution. The first 1,000 trees were discarded as burn-in to compute the consensus tree. We repeated the analyses three times to ensure the posterior probabilities were stable. Trees were rooted with a sequence of the promoter sequence of the *Sm*PoMuc pseudogene group 4.

### Sequence variation and gene diversity

We used DnaSP to characterise promoter sequence variation within and between groups of *Sm*PoMuc promoter sequences as the number of polymorphic sites, number of mutations between strains, net number of substitutions per site between strains and between groups of *Sm*PoMuc promoter sequences.

### Sequence variation of the promoter region of a single copy gene, *Sm*FTZ-F1, between *S. mansoni* IC and C strains

We amplified and sequenced the promoter region of the *Sm*FTZ-F1 gene. This gene encodes the nuclear receptor fushi tarazu-factor 1alpha and its promoter has been fully characterised [24] in 1 and 2 individuals of *S. mansoni* strains IC and C, respectively, from genomic DNA with primers *Sm*ftzf1-F (5′-ATGAGATGTTTCTGAGCAATGGC-3′) and Smftzf1-R (5′-TCTTCTCGTAGCTGAATCTGACC-3′) using the Advantage 2 PCR Enzyme System (Clontech). PCR amplicons were then sequenced and analysed for sequence variation and gene diversity as described above.

### Heterologous expression of promoter regions of *Sm*PoMuc genes

#### Cell culture and transfection

HeLa cells were maintained in Dulbecco's modified Eagle's medium (DMEM) and 10% fetal calf serum (FCS) containing an antibiotic/antimycotic mixture (penicillin 100 units/ml, streptomycin 0.1 mg/ml, amphotericin B 0.25 µg/ml; Sigma) at 37°C. Transfections were performed on Lab-Tek chamber slides (0.8 cm^2^/wells) with 250 ng of DNA using jetPRIME according to the manufacturer's instruction (Polyplus transfection). Briefly, 20,000 cells were seeded per well in 350 ml of cell growth medium 24 h prior to transfection. 250 ng of plasmid DNA diluted into 25 ml jetPRIME buffer were incubated with 1 ml jetPRIME transfectant for 10 min at room temperature. The transfection mix was added directly to the cells. After 72 h, we washed HeLa cells with PBS and fixed in −20°C methanol for 5 min. Cells were washed twice with PBS and counterstained with DAPI (100 mg/ml) for 10 sec and mounted with fluorescent mounting medium (Dako). Fluorescence was observed with a Zeiss Axioskop2 (Zeiss) using a camera Leica DC350FX coupled to imaging software (Leica FW4000).

### *Sm*PoMuc promoter construction

We amplified 996 kb of the *Sm*PoMuc group 3.1(r1–r2) promoter and 1002 kb of the *Sm*PoMuc group 3.1 promoters. These sequences are located just upstream of the transcriptional start site and have been amplified from the IC strain. These sequences were amplified using primers containing *Sac*I and *Bam*HI restriction sites (Table S2). The PCR product was gel-purified (Wizard SV gel and Clean-Up system,Qiagen), digested with both restriction enzymes and cloned into a *Sac*I and *Bam*HI digested pEGFP-1 reporter vector with T4 DNA ligase (New England Biolabs). The construct was verified by sequencing both DNA strands. Plasmids pEGFP-1 and pCMV-EGFP driving EGFP expression, under the control of the CMV-promoter, were used as negative and positive controls in the transfection assay.

### Sequence variation of promoter regions of *Sm*PoMuc group 1 gene between *S. mansoni* IC and C strains at the population level

A 3.3 kb region of the *Sm*PoMuc group 1 gene promoter region was amplified using primers SmpomucpromGP3.1.f2 and BR2 (Table S1) in individuals of each of *S. mansoni* IC and C strain. The PCR products span from 1.8 kb upstream of the TSS to the first repeat unit of the *Sm*PoMuc gene and cover the promoter region. 1.4 kb of the promoter region was sequenced for 20 and 18 individuals of the IC and C strains, respectively, by primer walking (Table S2). We used Arlequin 3.1 to characterise *Sm*PoMuc group 1 promoter diversity within the two strains as the expected unbiased gene diversity, the nucleotide diversity, corrected for sample size and incorporating nucleotide information [62]. We tested for sequence variation between the two strains using population comparisons and differentiation in Arlequin 3.1. Estimations incorporated Tamura-Nei distances between sequences and allele frequencies (Nei's *Φ*-estimator of *F_ST_*). The significance of genetic differentiation was tested by permuting the alleles among all samples 2,000 times. We also estimated the inbreeding coefficient in each strain using *f* and genetic differentiation between the two strains using *F_ST_* estimator *θ* ([63], incorporating allele frequencies only). Inbreeding coefficients and genetic differentiation for departure from the null hypothesis (*f* = 0, *θ* = 0) were tested using 2,000 permutations in GENETIX 4.05 [64].

### Allelic variation of 14 microsatellite loci between *S. mansoni* IC and C strains at the population level

Nineteen individuals of each of the IC and C strains were genotyped using 14 microsatellite loci [25]. We estimated genetic diversity of microsatellite loci as the mean number of alleles per locus (*A*) and observed and expected unbiased heterozygosities (*H_O_* and *Ĥ*? respectively) under the assumption of Hardy–Weinberg equilibrium [62]. We estimated the inbreeding coefficient *f* in each strain, genetic differentiation between the two strains *R_ST_* estimator [65], [66] and the *F_ST_* estimator *θ* as above.

### Trichostatin-A treatment, mRNA extraction, cDNA synthesis and transcription analysis

Trichostatin-A (TSA) (invivoGen met-tsa-5) was dissolved in ethanol to 20 mM and added to the 1000 IC or C miracidia pool at 20 µM and 200 µM during 4 h. We had shown previously the effect of TSA at these concentrations on development, morphology, mobility and gene expression without any cytotoxicity for the larvae [13], [27]. To the untreated control, an equal volume of ethanol was added (mock treatment). After 4 h, metamorphosis arrest was observed for larvae treated with TSA at 200 µM as expected for a positive effect with this drug [27]. Miracidia were then spun down at 12,000 g during 5 min and suspended in 100 µl of lysis buffer (Dynabeads mRNA DIRECT Micro kit, Dynal Biotech) in RNase-free tubes and stored at −80°C. Messenger RNAs were extracted using the Dynabeads mRNA isolation Kit according to the manufacturer's instructions. mRNA poly-A residues were eluted from the surface of the paramagnetic beads by a final denaturation step of 10 min at 75°C in 20 µl of Tris-HCl 10 mM. cDNA synthesis was carried out using 10 µl of mRNA in a final volume of 20 µl according to manufacturer's instructions (0.5 mM dNTPs, 0.01 mM DTT, 1× first strand buffer, 2 U RNase out, 10 U SuperScript II RT (Invitrogen) during 50 min at 42°C). After reverse transcription, the cDNAs were purified with the PCR clean-up system (Promega) and eluted into 100 µl 10 mM Tris/HCl (ph 7.5).

Specific primers for qPCR from groups 1, 2 and 3.1(r1–r2) were designed based on sequence alignment performed on cDNA variant representative of each group (Table S2). Their specificity was tested using as template a plasmid in which a cDNA variant of group 1, 2 or 3.1(r1–r2) was cloned. Group 4 genes contain a STOP codon in exon 8 of the gDNA sequence and their cDNA has never been detected. Therefore, transcripts of the group 4 genes were not targeted in this study. Other subgroups were not studied as it was not possible to design specific primers to amplify them. qPCR amplifications were performed as described below. Results were normalised with the αTub gene. The 2^ΔCt^ value was calculated. Statistical tests were performed on at least 3 different biological samples.

### Chromatin status of *Sm*PoMuc promoters by ChIP-qPCR

Native chromatin immunoprecipitation was performed as described before [67]. Briefly, antibodies against histone isoforms were used to precipitate chromatin in miracidia from IC and C strains (Table S3). DNA was extracted from the precipitated complex and analysed by qPCR using specific primers of *Sm*PoMuc groups 1, 3.1 and 3.1(r1–r2). Primers specifically targeting these genes were designed based on sequence alignment of *Sm*PoMuc promoter sequences (Table S2). We tested their specificity using as templates plasmids with promoters of group 1, 3.1 or 3.1(r1–r2). It was not possible to design primer sets that would hybridize specifically to the promoter sequences of the other groups or subgroups because conservation in the sequences resulted in cross-amplification between these groups. The amount of target DNA recovered in the immunoprecipitated fraction was quantified by calculating the percent input recovery (% IR) normalised with the percent input recovery obtained with a reference locus (αTub) as previously described [67].

### Chromatin status of *Sm*PoMuc promoter region by bisulfite treatment

Bisulfite genomic sequencing was carried out as described in [68]) on gDNA extracted from miracidia from the NMRI strain. Amplification was performed using primers BS.IC-1-Group1/1111-1715.48f GATATGTTTTAAGAAGTAGAAAAGAATATT, BS.IC-1-Group1/1111-1715.508r ATAAAAATTTTACAACCACCTACTC and BS.IC-1-Group3.1/421-952.29f ATTGTTTTTTTTAATTTTAGATATGTTTTA and two rounds of PCR. 1 µl of each PCR products were cloned into the TOPO TA vector (Invitrogen) and sequenced. *In-vitro* methylation with M.SssI (NEB) was done as recommended by the supplier. A total of 20 sequences (7 M.SssI treated positive controls and 13 target miracidial gDNA) were aligned with the genomic sequence from GenBank (Bioedit) to visualise the sites of methylated cytosine.

### qPCR analysis

qPCR amplifications were performed with 2.5 µl of immunoprecipitated DNA or cDNA in a final volume of 10 µl on a LightCycler® 480 II Real Time instrument (1.5 µl H_2_0, 0.5 µM of each primer, 5 µl of master mix). The following protocol was used: denaturation, 95°C for 10 minutes; amplification and quantification (40 times): 95°C for 10 seconds, 60°C for 10 seconds, 72°C for 20 seconds; melting curve, 65–97°C with a heating rate of 0.11°C/s and continuous fluorescence measurement, and a cooling step to 40°C. For each reaction, the cycle threshold (Ct) was determined using the “2nd derivative” method of the LightCycler® 480 Software release 1.5. PCR reactions were performed in duplicate and the mean value of Ct was calculated. Correct melting curves were checked using the Tm calling method of the LightCycler® 480 Software release 1.5. The amplification of a unique band was verified by electrophoresis separation through a 2% agarose gel for each qPCR product.

### GenBank accession numbers

JQ615951–JQ615966.

## Supporting Information

Figure S1 **Expression of each** ***Sm*** **PoMuc groups in C and IC strains and TSA effect.** mRNA were extracted from miracidia pool from the IC (Panel A) and C (panel B) strain and qPCR were performed with primers targeting *Sm*PoMuc group 1, group 2, group 3.1(r1–r2). The results of 3 experiments are represented on each graph (Experiment 1: Black bars, Experiment 2: Dark grey bars, Experiment 3: pale grey bars). A Friedman non-parametrical test was performed to test the significance of the increase of expression after TSA was added. The p value of the Friedman test is indicated on each graph.
(TIF)

Figure S2 **Immunoprecipitation of miracidia chromatin: Comparison of the chromatin state of the different group within a strain.** ChIP experiments were performed on chromatin isolated from miracidia from both the IC and C strain with antibodies that target H3 acetylated on lysine 9 and H3 tri-methylated on lysine 9. Immunoprecipitated chromatin was analysed by qPCR using primers that target specific sequences of *Sm*PoMuc group 3.1(r1–r2), 1 and 3.1. Results represent the percentage input recovery (%IR) normalised with %IR from a reference gene (αTub). Results are the average of 3 biological repeats. All p value from t-test that compare the results obtained with group 3.1(r1–r2) and group 1, group 3.1(r1–r2) and group 3.1, group1 and group 3.1 are below 0.05 in the IC strain for both antibodies.
(TIF)

Figure S3 **Immunoprecipitation of chromatin from miracidia, cercaria and adults over 3 generations.** ChIP was performed on chromatin isolated from IC strain miracidia (Black bars), cercaria (grey bars) and adults (white bars). ChIP was performed with antibodies against H3 acetylated on lysine 9 (panel A) and H3 tri-methylated on lysine 9 (H3K9Met3). Immunoprecipitated chromatin was analysed by qPCR using primers that hybridize with specific sequences of *Sm*PoMuc group 3.1(r1–r2), group 3.1and group 1. Results represent the percentage input recovery (%IR) on target gene normalised with % IR of a reference gene (αTub) obtained on 3 generations (G1, G2, G3). Results are the average of 2 technical repeats.
(TIF)

Table S1 **Origin of the sequences used for phylogenetic analysis of** **fig. 3** **.**
(DOCX)

Table S2 **Primers used in this study.**
(DOCX)

Table S3 **Antibodies used in this study.**
(DOCX)
