# Supplementary figures and images for: Schistosoma mansoni Mucin Gene (SmPoMuc) Expression: Epigenetic Control to Shape Adaptation to a New Host
Source: PLoS Pathog. 2013 Aug 29;9(8):e1003571. doi: 10.1371/journal.ppat.1003571 (PMC3757033; doi:10.1371/journal.ppat.1003571)

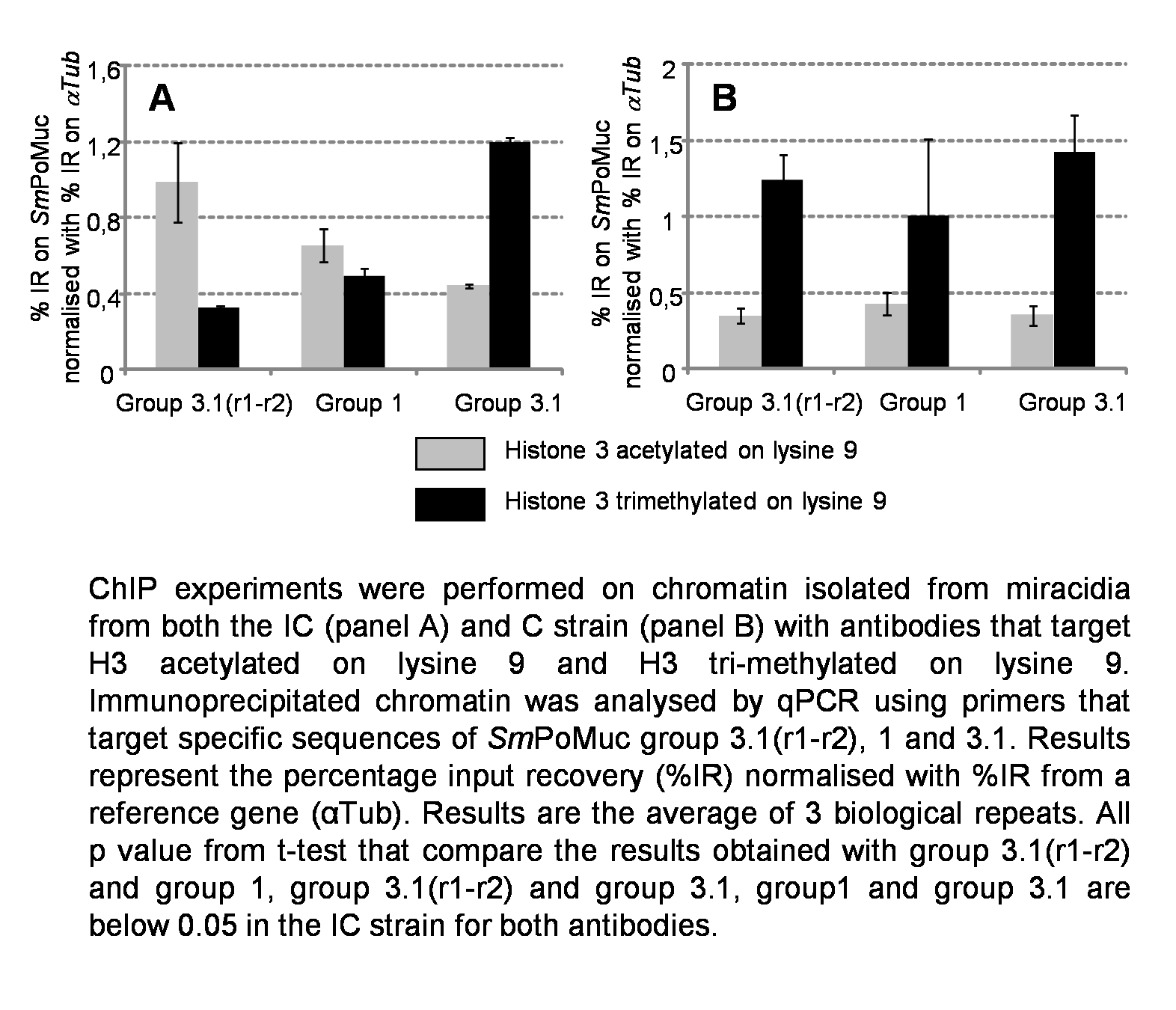

Supplement: Figure S2 — Immunoprecipitation of miracidia chromatin: Comparison of the chromatin state of the different group within a strain. ChIP experiments were performed on chromatin isolated from miracidia from both the IC and C strain with antibodies that target H3 acetylated on lysine 9 and H3 tri-methylated on lysine 9. Immunoprecipitated chromatin was analysed by qPCR using primers that target specific sequences of SmPoMuc group 3.1(r1–r2), 1 and 3.1. Results represent the percentage input recovery (%IR) normalised with %IR from a reference gene (αTub). Results are the average of 3 biological repeats. All p value from t-test that compare the results obtained with group 3.1(r1–r2) and group 1, group 3.1(r1–r2) and group 3.1, group1 and group 3.1 are below 0.05 in the IC strain for both antibodies. (TIF) [file ppat.1003571.s002.tif]

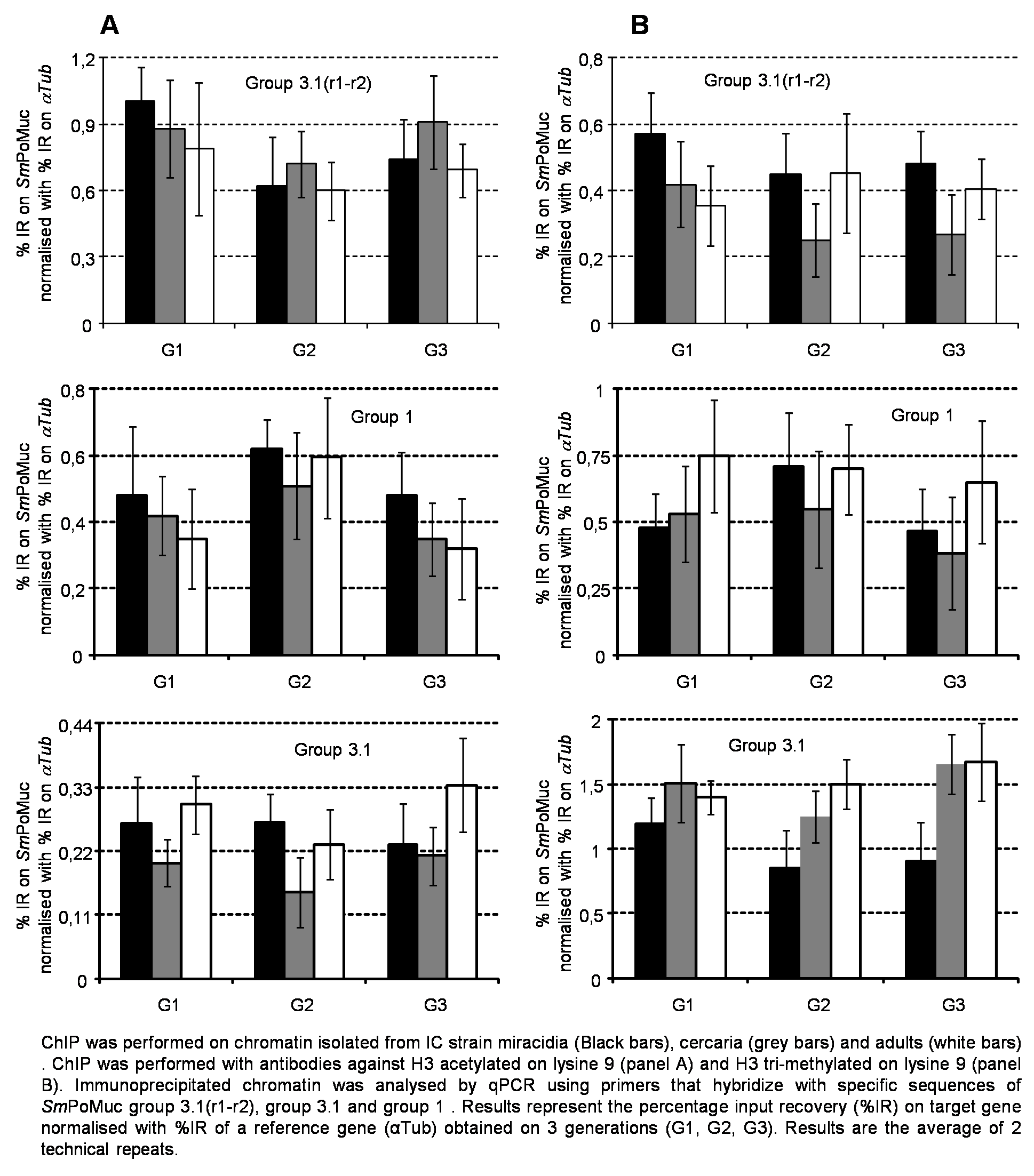

Supplement: Figure S3 — Immunoprecipitation of chromatin from miracidia, cercaria and adults over 3 generations. ChIP was performed on chromatin isolated from IC strain miracidia (Black bars), cercaria (grey bars) and adults (white bars). ChIP was performed with antibodies against H3 acetylated on lysine 9 (panel A) and H3 tri-methylated on lysine 9 (H3K9Met3). Immunoprecipitated chromatin was analysed by qPCR using primers that hybridize with specific sequences of SmPoMuc group 3.1(r1–r2), group 3.1and group 1. Results represent the percentage input recovery (%IR) on target gene normalised with % IR of a reference gene (αTub) obtained on 3 generations (G1, G2, G3). Results are the average of 2 technical repeats. (TIF) [file ppat.1003571.s003.tif]
