# Supplementary material for: Schistosoma mansoni Mucin Gene (SmPoMuc) Expression: Epigenetic Control to Shape Adaptation to a New Host
Source: PLoS Pathog. 2013 Aug 29;9(8):e1003571. doi: 10.1371/journal.ppat.1003571 (PMC3757033; doi:10.1371/journal.ppat.1003571)
Supplement: Table S1 — Origin of the sequences used for phylogenetic analysis of fig. 3 . (DOCX) [file ppat.1003571.s004.docx]

Table S1: Origin of the sequences used for phylogenetic analysis of fig. 3.

| Genbank Accession Number | Target | DNA template | Sequence  length | Primer Name and Sequence to generate PCR products | Amplicon  size |
| --- | --- | --- | --- | --- | --- |
| Group 1 | | | | | |
| JQ615951 | IC-1 | gDNA | 1.8kb | SmpomucpromGP3.1.f2: 5’-GCCTGCATAAATAGGGACCG-3’ | 3.3kb |
| JQ615952 | C-1 | gDNA | 1.8kb | BR2: 5’-AACTCACCTGTGGGTTTGTCTG-3’ |  |
| Group 2 | | | | | |
| JQ615953 | IC-1 | Phage library^b^ | 1.74kb | Smpomuc2f3: 5’-TGTTGGATGTTTATATCTATGC-3’ | 2.4kb |
| JQ615954 | IC-2 | gDNA | 1.74kb | exon1R: 5’-AGAGAATAATTTTCTTGTTCATTCTTC-3’ |  |
| JQ615955 | C-1 | gDNA | 1.74kb |  |  |
| JQ615956 | NMRI-1 |  | 1.74kb | Sequence obtained from assembled sequences BACs 41B11, 62F12^a^ | |
| Group 3.1 | | | | | |
| JQ615957 | IC-1 | gDNA | 1.04kb | smpomuc2f2: 5’-GGAATAGTCTAGTGATAACG-3’ | 9.0kb |
| JQ615958 | C-1 | gDNA | 1.04kb | exon1R: 5’-AGAGAATAATTTTCTTGTTCATTCTTC-3’ |  |
| JQ615959 | C-2 | gDNA | 1.04kb |  |  |
| JQ615960 | NMRI-1 | BAC47P6^a^ | 1.04kb |  |  |
| JQ615961 | NMRI-2 | BAC47P6^a^ | 1.04kb |  |  |
| JQ615962 | NMRI-3 |  | 1.04kb | Sequence obtained from database assembly version 3.1 | |
| Group 3.1 (r1-r2) | | | | | |
| JQ615963 | IC-1 | gDNA | 2.00kb | exon2f : 5’-TTCTTAGCACTACCCAAAGATGAAC-3’ | 9.0kb |
|  |  |  |  | exon1R: 5’-AGAGAATAATTTTCTTGTTCATTCTTC-3’ |  |
| JQ615964 | C-1 | gDNA | 2.00kb | Prom groupe4 spe2.for : 5’-TTTCATCATTGTGCGGTGTT-3’ | 3.0kb |
|  |  |  |  | prom univ3.rev : 5’-GCCTAATTTTAATTCACAATCTACTCA-3’ |  |
| JQ615965 | NMRI-1 |  | 2.00kb | Sequence obtained from database assembly version 3.1 | |
|  |  |  |  |  | |

^a^ The BAC library was previously described by Roger et al. 2008b.

^b^ The phage library was constructed in this work and is described in the materials and methods section.
