# Supplementary material for: Schistosoma mansoni Mucin Gene (SmPoMuc) Expression: Epigenetic Control to Shape Adaptation to a New Host
Source: PLoS Pathog. 2013 Aug 29;9(8):e1003571. doi: 10.1371/journal.ppat.1003571 (PMC3757033; doi:10.1371/journal.ppat.1003571)
Supplement: Table S2 — Primers used in this study. (DOCX) [file ppat.1003571.s005.docx]

Table S2: Primers used in this study

Table 1. *Sm*PoMuc primers used for sequencing and primer-walking.

| Target | Name | Primer sequence |
| --- | --- | --- |
| Promoter group 1 | smpomucpromGP3.1.f2 | 5’-GCCTGCATAAATAGGGACCG-3’ |
|  | BR2 | 5’-AACTCACCTGTGGGTTTGTCTG-3’ |
|  | smpomucprom1.r1 | 5’-TCACATCCGTGACACTTTCAG-3’ |
|  | smpomucprom.r1 | 5’-TGGACACACGGTAAATAAAACTG-3’ |
|  | smpomucprom.r12 | 5’-TCGGGTGGCATTTTCATCACGG-3’ |
|  | smpomucprom1.r2 | 5’-CTGAAATCCCGGAAGAACTG-3’ |
| Promoter group 2 | smpomuc2.f3 | 5’-TGTTGGATGTTTATATCTATGC-3’ |
|  | exon1R | 5’-AGAGAATAATTTTCTTGTTCATTCTTC-3’ |
|  | smpomucprom.f1 | 5’-CTACGAGTAGGTGGCTGCAA-3’ |
|  | smpomucprom4.r1 | 5’-AATGTGCACGAAACATGGAA-3’ |
| Promoter group 3.1 | smpomuc2.f2 | 5’-GGAATAGTCTAGTGATAACG-3’ |
|  | exon1R | 5’-AGAGAATAATTTTCTTGTTCATTCTTC-3’ |
|  | Smpomucprom.f1 | 5’-CTACGAGTAGGTGGCTGCAA-3’ |
|  | smpomucprom.r12 | 5’-TCGGGTGGCATTTTCATCACGG-3’ |
|  | smpomucprom.r3 | 5’-TTTATATTCGTGATTTCCCTGTTC-3’ |
| Promoter group 3.1(r1-r2) | exon2f | 5’-TTCTTAGCACTACCCAAAGATGAAC-3’ |
|  | exon1R | 5’-AGAGAATAATTTTCTTGTTCATTCTTC-3’ |
|  | smpomucprom.r1 | 5’-TGGACACACGGTAAATAAAACTG-3’ |
|  | smpomucprom.r2 | 5’-TGGTGCAAATGAAAAGCGTA-3’ |
|  | smpomucprom4.r1 | 5’-AATGTGCACGAAACATGGAA-3’ |

Table 2. Primers used for phage DNA screening

|  | Name | Primer sequence | Discrimination criteria |
| --- | --- | --- | --- |
| Primer set 1 | repetB.F2 | 5’-TCTCACATTTCAGGTGACCTC-3’ | Group 1expected size : 1150bp |
|  | Intron3/4R | 5’-CACATGCATAGCTAATGTGGTAATG-3’ | Group 2 expected size : 1727bp |
|  |  |  | Group 3 and 4: no amplification expected |
| Primer set 2 | repetG.F2 | 5’-GCTCTCACATTTCAGATGACTAT-3’ | Group 3.1 expected size: 1136bp |
|  | Intron3/4R | 5’-CACATGCATAGCTAATGTGGTAATG-3’ | Group 1, 2, 4, 3.3, 3.4: no amplification expected |
| Primer set 3 | URnew | 5’-GTGGCCATGTAACAAACGCACC-3’ | Group 4 expected size: 1150bp |
|  | Intron3/4R | 5’-CACATGCATAGCTAATGTGGTAATG-3’ | Group 1, 2, 3: no amplification expected |
| Primer set 4 | repetB.F2 | 5’-TCTCACATTTCAGGTGACCTC-3’ | Specific for the presence of combinatory event |
|  | repetG.R2 | 5’-AACTCACCTGTTGGTTCGCTC-3’ |  |

Table 3. Primers used in this study for transcription and ChIP analysis by qPCR

| Target | Name | Primer sequence | size (pb) | Tm | Primer efficiency | Specificity |
| --- | --- | --- | --- | --- | --- | --- |
| Primer sets for expression analysis by qPCR | | | | | | |
| cDNA group 1 | *Sm*PoMuc1.E8/9.F | 5’-CGAAAGTGCTTACATCGCTG-3’ | 208 | 68°C | 1.93 | All group 1 |
|  | *Sm*PoMuc1.E12.R | 5’-CGAATAGGCTTCTTTATGTTG-3’ |  |  |  |  |
| cDNA group 2 | *Sm*PoMuc2.E4.F | 5’-CCAGATCCACAACAGCACC-3’ | 260 | 68°C | 2 | group 2 (exclude truncated group 2) |
|  | *Sm*PoMuc2.E8.R | 5’-ACTTTCGACGTCATCAAGAAG-3’ |  |  |  |  |
| cDNA group 3.1(r1-r2) | URG/B.F | 5’- CGAACCAACAGGTGACCTCGC-3’ | ND | 65°C | 2 | Specific to subgroup 3.1 with combinatorial event of tandem repeats^a^ |
|  | *Sm*PoMuc3 E2/E3.R | 5’-CTGGAACTGTTGGTTCGCTC-3’ |  |  |  |  |
| Primer sets for ChIP analysis by qPCR | | | | | | |
| Promoter group 3.1(r1r2) | prom-group3.1(r1-r2).for | 5’-ATGATTTTACGAGAGGTTCAGT-3’ | 101 | 60°C | 2 | Specific to promoter of subgroup 3.1 with combinatorial event of tandem repeats^b^ |
|  | prom-group3.1(r1-r2).rev | 5’-ggtgcactattcagcctagtca-3’ |  |  |  |  |
| Promoter group 1 | prom-group1-spe4.for | 5’-TCTCTACTGAAAGTGTCACGGA-3’ | 130 | 60°C | 1.96 |  |
|  | prom-group1-spe4.rev | 5’-TGAGTGAAGGTTTTGCAGCC-3’ |  |  |  |  |
| Promoter group 3.1 | prom-group3.1-spe2.for | 5’ATACGAGTCAGTATTAGTAAATG-3’ | 100 | 60°C | 1.91 |  |
|  | prom-group3.1-spe2.rev | 5’-TTTATCGATCGTAACTTTGGAC-3’ |  |  |  |  |

^a^ Previous studies have shown that *Sm*PoMuc genes contain 3 kinds of tandem repeats in their 5’ region. Two of them, r1 and r1′, display only 1 nucleotide and 1 amino acid difference (Roger et al. 2008b). The third repeat r2 is highly divergent. The sub-group 3.1 is represented by several copies in the genome. One of these copies (and only in this sub-group 3.1) contains the combination of r1 and r2 type of repeats in their 5’ region in both the C and IC strain but expression of the intermingled repeat units is a specific feature of the IC strain (Roger et al. 2008b). In this study, primer sets designed to target the group 3.1 are specific to this particular *Sm*PoMuc cDNA that contains the intermingled repeat units.

^b^ The promoter sequence of this group 3.1 was identified as the promoter of the sub-group 3.1 that contains the intermingled repeat units. This was demonstrated by PCR analysis on a phage of the phage lambda library that contains the sub-group 3.1 (r1-r2) as an insert.

Table 4. Primers used to amplify promoter sequence for heterologous expression analysis

|  | Name | Primer sequence | Size (bp) |
| --- | --- | --- | --- |
| *Sm*PoMuc group 3.1 JQ615957 | prom-group3.1exp.for (position 6 on JQ615957) | 5’-CCCTTTgagctcATGGAGCAGTGAGTTA-3’ | 1002 |
|  | prom-group3.1exp.rev (position 1007 on JQ615957) | 5’-TATggatccTGGCTTTATATATATTTTTAC-3’ |  |
| *Sm*PoMuc group3.1(r1-r2) JQ615963 | prom-group3.1(r1-r2)exp.for (position 975 on refseq JQ615963) | 5’-AATTCgagctcACATGGTTTTATATACGTATTGTCTTC-3’ | 996 |
|  | prom-group3.1exp.rev (position 1970 on JQ615963) | 5’-TATggatccTGGCTTTATATATATTTTTAC-3’ |  |
