# Supplementary material for: Schistosoma mansoni Mucin Gene (SmPoMuc) Expression: Epigenetic Control to Shape Adaptation to a New Host
Source: PLoS Pathog. 2013 Aug 29;9(8):e1003571. doi: 10.1371/journal.ppat.1003571 (PMC3757033; doi:10.1371/journal.ppat.1003571)
Supplement: Table S3 — Antibodies used in this study. (DOCX) [file ppat.1003571.s006.docx]

Table S3: Antibodies used for N-ChIP.

| Antibody | Host | Product | Lot | Saturating quantity used for N-ChIP |
| --- | --- | --- | --- | --- |
| H3K9Ac | Rabbit | Upstate, 07352 | DAM1576933 | 8μl |
| H3K9Met3 | Rabbit | Abcam, Ab8898 | 733951 | 4μl |
| H3K4Met3 | Rabbit | Upstate, 04745 | JBC1355340 | 4μl |
